# Lifespan Intellectual Factors, Genetic Susceptibility, and Cognitive Phenotypes in Aging: Implications for Interventions

**DOI:** 10.3389/fnagi.2019.00129

**Published:** 2019-05-31

**Authors:** Yongxiang Wang, Yifeng Du, Juan Li, Chengxuan Qiu

**Affiliations:** ^1^Department of Neurology, Shandong Provincial Hospital Affiliated to Shandong University, Jinan, China; ^2^Center on Aging Psychology, Institute of Psychology, Chinese Academy of Sciences, Beijing, China; ^3^Department of Psychology, University of Chinese Academy of Sciences, Beijing, China; ^4^Department of Neurobiology, Care Sciences and Society, Aging Research Center and Center for Alzheimer’s Research, Karolinska Institutet-Stockholm University, Stockholm, Sweden

**Keywords:** psychosocial factors, genetic susceptibility, interaction, cognitive aging, cognitive reserve, life-course epidemiology

## Abstract

Along with rapid global population aging, the age-related cognitive disorders such as mild cognitive impairment (MCI) and dementia have posed a serious threat to public health, health care system, and sustainable economic and societal development of all countries. In this narrative review, we seek to summarize the major epidemiological studies from the life-course perspective that investigate the influence of genetic susceptibility [e.g., apolipoprotein (APOE) ε4 allele] and intellectual or psychosocial factors (e.g., educational attainments and leisure activities) as well as their interactions on cognitive phenotypes in aging. Numerous population-based studies have suggested that early-life educational attainments and socioeconomic status, midlife work complexity and social engagements, late-life leisure activities (social, physical, and mentally-stimulating activities), certain personality traits (e.g., high neuroticism and low conscientiousness), and depression significantly affect late-life cognitive phenotypes. Furthermore, certain intellectual or psychosocial factors (e.g., leisure activities and depression) may interact with genetic susceptibility (e.g., APOE ε4 allele) to affect the phenotypes of cognitive aging such that risk or beneficial effects of these factors on cognitive function may vary by carrying the susceptibility genes. Current evidence from the randomized controlled trials that support the cognitive benefits of cognitive training among cognitive healthy older adults remains limited. The cognitive reserve hypothesis has been proposed to partly explain the beneficial effects of lifetime intellectual and psychosocial factors on late-life cognitive function. This implies that, from a life-course perspective, preventive intervention strategies targeting multiple modifiable intellectual and psychosocial factors could interfere with clinical expression of cognitive disorders in old age and delay the onset of dementia syndrome, and thus, may help achieve healthy brain aging.

## Introduction

Cognitive aging can be referred to as a process of gradual deteriorations in cognitive function that occur as people age. Phenotypes of cognitive aging as a continuum range from normal age-related cognitive decline through mild cognitive impairment (MCI) to a full stage of dementia; Alzheimer’s disease is the most common form of dementia among older people. The global aging population has increased steadily, especially in low- and middle-income countries such as China and India (Chatterji et al., 2008; Zhang et al., 2012). As a result, the aging-related cognitive disorders such as MCI and dementia have posed tremendous challenges not only for current health care and social welfare system but also for sustainable socioeconomic development of all societies (Wimo et al., 2017; Xu et al., 2017). Dementia, as the most common dementing disorder in older people, is defined as a clinical syndrome characterized by a progressive deterioration in multiple cognitive domains (e.g., memory, attention, executive, and verbal fluency) that are severe enough to interfere with the functioning of daily life. MCI and dementia are the principal causes of functional dependence, poor quality of life, institutionalization, and mortality among older people. Thus, the accelerated cognitive deterioration in aging is the major hamper to achieve successful aging and longevity (Depp et al., 2012). The overall prevalence of dementia among people aged 60 years or older is estimated to be about 6%–8%, and after 60 years of age, the likelihood of dementia occurrence almost doubles every 5–7 years (Prince et al., 2013; Winblad et al., 2016). It was estimated that in 2015, worldwide nearly 47 million people were living with dementia, and the number was projected to reach ~74.7 million by 2030 and ~131.5 million by 2050, with the global costs of dementia in 2015 being estimated at ~$818 billion (Wimo et al., 2017). MCI is even more prevalent than dementia, with the prevalence ranging from 10% to 20% (Petersen et al., 2010; Nie et al., 2011). Since the 1980s, numerous community-based cohort studies of older people (e.g., age ≥60 years) focusing on cognitive aging and dementia have been implemented across the world, especially in Europe and North America (Stanziano et al., 2010). In addition, several observational studies that were initiated among young and middle-aged people in the 1960s and the 1970s have shifted their initial focus from metabolic and cardiovascular disease (e.g., obesity, hypertension, diabetes, and ischemic heart disease) to phenotypes of cognitive aging (e.g., MCI and dementia) as the study cohorts age (Irie et al., 2008; Håkansson et al., 2009; Chang et al., 2010). These long-term observational studies have significantly contributed to the better understanding of multifactorial etiology and the process of cognitive aging and dementia from the life-course perspective (Whalley et al., 2006; Fratiglioni and Qiu, 2011; Qiu, 2012). Whereas it has now been well-established that dementia and cardiovascular disease share common cardiometabolic risk factors from midlife onward, increasing evidence supports the potential role of the life-course intellectual factors (e.g., education, work complexity, and mental activity) in delaying the onset of cognitive disorders (e.g., dementia). In this brief narrative review, we sought to summarize evidence from the systematic reviews and the major up-to-date epidemiological studies concerning the effects of genetic susceptibility, intellectual factors over the lifespan, and their interactions on cognitive phenotypes in old age (e.g., cognitive decline, MCI, and dementia) as well as potential implications for interventions.

## The Life-Course Approach in Cognitive Aging

The life-course approach considers biological, physical, and psychosocial environmental factors acting over the lifespan (e.g., gestation, early childhood, adolescence, young adulthood, midlife, and older age) to be relevant for determining the risk of chronic diseases (e.g., cancer, cardiovascular disease, and dementia) occurring later in life (Ben-Shlomo and Kuh, 2002). This approach seeks to identify specific time-windows over the lifespan when exposures have the greatest effect on health outcomes and to determine whether accumulative exposures over the lifespan have interactive (e.g., synergistic, additive or multiplicative) effects on health outcomes in late life (Whalley et al., 2006; Richards and Hatch, 2011). Thus, from the life-course perspective, the late-life cognitive phenotypes are not determined by exposures in any single time-period over the lifespan; rather, they reflect the result of complex interactions of genetic susceptibility, biological factors, and psychosocial environments experienced over the whole life. For instance, the cognitive reserve hypothesis, which is proposed to interpret the disparities between clinical phenotypes of cognitive aging and the load of neuropathologies in the brain (Stern, 2012), can be conceived as the sum of lifetime input of cognitive reserve or intellectual factors (Richards and Deary, 2005; Dekhtyar et al., 2015, 2016). As an example in understanding the etiology of a chronic disease from a life-course perspective, evidence has emerged that late-life risk of dementia is determined by genetic susceptibility and life-long exposures to non-genetic physical, biological, and psychosocial environmental factors as well as their interactions (Qiu, 2012). Furthermore, the life-course model introduces the concept of time window at exposure that is highly relevant for studying the etiology or determinants of chronic diseases with a long-term latent period such as dementia. A factor that increases the risk of a chronic disease if the exposure occurs in a certain time-period may show a differential effect if it occurs in another time-period over the lifespan, owing to various psychosocial and biological mechanisms, interactions with other factors, or selective survival. For instance, using the life-course approach, systematic reviews have identified specific time-windows for certain cardiometabolic risk factors (e.g., high blood pressure, obesity, and high cholesterol) that act as risk factors for late-life cognitive impairment and dementia, mainly when they occur in young adulthood and middle age, but not necessarily in late-life (Qiu et al., 2005; Qiu and Fratiglioni, 2015; Irwin et al., 2018). In this context, it is important to keep the life-course perspective in mind when designing intervention programs to delay clinical expression of cognitive disorders in old age.

Notably, the meta-analysis of population-based neuropathological imaging studies has revealed a 20- to 30-year interval between the first deposit of hallmark Alzheimer pathology in the brain (i.e., amyloid) and the onset of the first clinical symptoms of dementia (Jansen et al., 2015). This implies that owing to the relatively long-term latent (pre-clinical) period of dementia, the potential reverse causality should be kept in mind when interpreting the associations between exposures to environmental factors and the risk of dementia even from the prospective cohort studies.

## Genetic Susceptibility

Mutations in amyloid precursor protein, presenilin-1, and presenilin-2 genes cause early-onset familial Alzheimer’s disease, but such cases only account for <3% of all Alzheimer cases (Qiu et al., 2009; Ballard et al., 2011). Late-onset sporadic Alzheimer’s disease accounts for the large majority of Alzheimer cases, which is determined by genetic and environmental factors as well as their interactions over the life-course.

Apolipoprotein E (APOE) ε4 allele is so far the only established genetic factor for sporadic Alzheimer’s disease. The onset age of Alzheimer’s disease is decreased by about 3–4 years for people who carry every APOE ε4 allele (Sando et al., 2008). The risk of Alzheimer’s disease increases with increasing number of the ε4 allele (Qiu et al., 2009), although the risky effect of APOE ε4 allele tends to decrease with advancing age. It is estimated that around 15%–20% of Alzheimer cases in the general elderly population are attributable to carrying the APOE ε4 allele. However, evidence supporting the association of APOE ε4 allele with MCI or cognitive impairment no dementia remains mixed. A meta-analysis suggests that APOE ε4 allele does affect cognitive function in normal aging, but the influence is relatively small and is specific to certain cognitive domains such as episodic memory and executive function (Small et al., 2004). Indeed, data from the Swedish Kungsholmen Project suggested that APOE ε4 allele was associated with global cognitive decline with subsequent progression to dementia, whereas the association with cognitive decline without progression to dementia was less evident (Qiu et al., 2006). Thus, APOE ε4 allele is associated primarily with cognitive decline owing to incipient dementia (Hayden et al., 2009; Qiu and Fratiglioni, 2010). This is in line with the meta-analysis suggesting that APOE ε4 allele is associated with a moderately increased risk for progression from MCI to Alzheimer dementia (Elias-Sonnenschein et al., 2011).

In addition, several other candidate genes that are often related to cardiometabolic risk factors have been associated with cognitive phenotypes in aging, such as angiotensin-converting enzyme (ACE) gene, cholesterol 24-hydroxylase gene, fat and obesity-associated FTO gene, and insulin degrading enzyme (IDE) gene (Guerreiro et al., 2012; Reitz et al., 2012; Schrijvers et al., 2012; Zettergren et al., 2017; Haithem et al., 2018). Furthermore, the genome-wide association studies have identified various susceptibility loci or variants that are potentially associated with an elevated risk of Alzheimer’s disease (Harold et al., 2009; Hollingworth et al., 2011; Lambert et al., 2013). However, their associations with late-life cognitive disorders are relatively weak, although a polygenic risk score outside the APOE ε4 locus may help improve risk prediction for MCI and Alzheimer’s disease (Chouraki et al., 2016; Logue et al., 2019).

## Intellectual or Psychosocial Factors

### Educational Attainments

Since the early 1990s when the Shanghai Aging Study linked illiteracy or low education with late-life sporadic dementia and Alzheimer’s disease (Zhang et al., 1990), numerous epidemiological studies have suggested that early-life low educational attainment is associated with an increased risk of late-life cognitive impairment and dementia, in which the association could not be explained by unhealthy lifestyle and low occupational position associated with low education (Karp et al., 2004; Ngandu et al., 2007; Qiu et al., 2010). The quantitative meta-analyses of prospective cohort studies reported that low education (middle school or below vs. high school or above) was associated with a ~60% increased risk of dementia (Caamaño-Isorna et al., 2006; Norton et al., 2014). A systematic review found that lower education was associated with a greater risk for dementia in many but not all studies and that the effect of early-life educational attainment on dementia risk may be best evaluated within the context of a lifespan developmental model (Sharp and Gatz, 2011). In addition, cognitive ability in childhood, intelligence quotient, and bilingualism also may postpone onset of cognitive impairment and dementia (McGurn et al., 2008; Craik et al., 2010). Furthermore, a higher socioeconomic position over the lifespan was associated with a lower risk of dementia later in life, suggesting that exposures to socioeconomic disadvantage contribute to late-life cognitive phenotypes (Marengoni et al., 2011; Zeki Al Hazzouri et al., 2011). Finally, when early-life educational attainment and occupation-based socioeconomic position were examined simultaneously in association with cognitive impairment and dementia, an independent association existed only with education (Karp et al., 2004), suggesting that education may play a predominant role over occupational position in determining late-life cognitive phenotypes.

### Occupational Complexity

Evidence from several population-based studies supports cognitive benefits of occupational complexity. The US Coronary Artery Risk Development in Young Adults study (age 18–30 years) showed that occupational cognitive complexity earlier in adulthood was associated with better white-matter integrity and performance in processing speed and executive function in midlife (Kaup et al., 2018). The Lothian Birth Cohort 1936 study suggested that complexity of work with people and data was associated with better cognitive performance later in life, independent of IQ, education, and social deprivation (Smart et al., 2014). The Swedish Twin Study and the Kungsholmen Project showed that a greater work complexity was associated with a reduced risk of dementia (Andel et al., 2005; Karp et al., 2009). Similarly, using a life-course model of different data sources, additional two Swedish studies suggested that high school performance in childhood and complex occupations in adulthood were both associated with a lower risk of dementia (Dekhtyar et al., 2015, 2016). Finally, the cognitive benefits of more occupational complexity and more leisure activities were also supported even in elderly populations with overall low educational attainments and low socioeconomic position (Darwish et al., 2018). The cognitive reserve hypothesis has been proposed to explain the cognitive benefits of adulthood work complexity (Stern, 2012).

### Physical, Social, and Mental Activity

#### Physical Activity

In 2004, a systematic review found that the majority of longitudinal studies supported an association of physical activity with a reduced risk of dementia (Fratiglioni et al., 2004). Since then, evidence has accumulated to support the potential cognitive benefits of physical activity. Regular physical exercise, even low-intensity activity such as walking, also was associated with a reduced risk of dementia and cognitive decline in older adults (Larson et al., 2006; Tomata et al., 2019). Long-term follow-up studies suggested that physical activity at any time point over the lifespan, especially in early life (e.g., teenagers) and middle age, was associated with a lower likelihood of late-life cognitive impairment and dementia (Andel et al., 2008; Taaffe et al., 2008; Middleton et al., 2010; Zotcheva et al., 2018; Palta et al., 2019). The Rush Memory and Aging Project showed that a higher level of daily physical activity was associated with a lower risk of dementia and global cognitive decline (Buchman et al., 2012). The systematic reviews of prospective studies revealed that physical activity during middle age and later in life might reduce the risk of dementia and cognitive impairment by about 35%–45% (Hamer and Chida, 2009), although the cognitive benefits owing to late-life physical activity may partially reflect a reverse causation (Morgan et al., 2012). A recent systematic review of prospective observational studies found that the majority of studies reported that leisure-time physical activity, but not work-related physical activity, was associated with a reduced risk of Alzheimer’s disease (Stephen et al., 2017). Theoretically, regular physical activity may promote vascular and circulatory health by reducing blood pressure, obesity, and blood glucose, although physical activity often contains components of social and cognitive activities, which may provide cognitive reserve. However, the Whitehall II study showed no evidence for the inverse association between midlife physical activity (age 35–55 years) and late-life risk of cognitive decline and dementia (Sabia et al., 2017). In addition, the Lifestyle Interventions and Independence for Elders Study concluded that a 2-year moderate-intensity physical activity program did not improve global or domain-specific cognitive function (Sink et al., 2015). Finally, the systematic reviews of randomized controlled trials concluded that evidence was largely insufficient that single-component physical activity or aerobic physical activity could prevent cognitive decline or dementia among cognitively healthy older adults (Young et al., 2015), and that multidomain interventions might delay cognitive decline (Brasure et al., 2018).

#### Social Engagement and Social Network

The systematic review of longitudinal studies in 2004 found evidence suggesting that social disengagement, a poor social network, and social isolation later in life are associated with an elevated risk of dementia (Fratiglioni et al., 2004). Similarly, the updated systematic reviews of longitudinal studies concluded that social relationship factors that represented a lack of social interactions were associated with an increased risk of dementia, in which the strength of the associations was comparable with other well-established risk factors for dementia, such as low educational attainment, physical inactivity, and late-life depression (Kuiper et al., 2015). Additionally, the French PAQUID cohort study showed that late-life social engagement was independently associated with a reduced risk of dementia (Marioni et al., 2015). Similarly, in the Honolulu-Asia Aging Study, low social engagement in late-life and a decline in social engagement from middle age to late-life were associated with a two-fold increased risk of dementia (Saczynski et al., 2006); the association was supported by systematic reviews (Penninkilampi et al., 2018). The Finnish CAIDE study found that being widowed from midlife onwards was associated with a substantial risk of dementia, suggesting that living with a partner might imply cognitive and social challenges that potentially protected against late-life dementia and cognitive decline (Håkansson et al., 2009). Finally, the US Health and Retirement Study suggested that late-life loneliness was associated with a 40% increased, whereas purpose in life was associated with a 30% decreased, risk of dementia independent of social isolation and other genetic and environmental factors (Sutin et al., 2018a,b). Taken together, cumulative evidence supports that more socially active older adults experience less cognitive decline and a reduced risk of dementia in late-life (James et al., 2011).

#### Mentally-Stimulating Activity

In the past decades, numerous population-based cohort studies have frequently reported that a greater engagement in intellectual or mentally-stimulating activities (e.g., learning, reading, handicrafts, doing crossword puzzle or playing games) from young adulthood through midlife and old age is associated with a lower risk of dementia, as summarized and concluded in several systematic reviews (Fratiglioni et al., 2004; Sajeev et al., 2016; Yates et al., 2016). Some studies showed that their association remains even when these activities are assessed more than 5 years prior to diagnosis of dementia (Akbaraly et al., 2009; Marioni et al., 2015), supporting a potential temporal relationship of intellectual activities to cognitive benefits. Cognitive reserve has been proposed to explain the observed cognitive benefits associated with cognitive or mental activities, although neuroimaging studies also suggested that cognitively engaging activities were associated with greater volumes of cortex and subcortex (e.g., brain reserve; Seider et al., 2016). However, the systematic reviews of randomized controlled trials or intervention studies found little or insufficient evidence that the short-term computerized cognitive training (e.g., ≥12 weeks) or even longer cognitive training exercises (≥6 months) could improve cognitive function or delay cognitive decline or onset of dementia among healthy older adults (Lampit et al., 2014; Butler et al., 2018; Gates et al., 2019). This may suggest that cognitive benefits suggested in observational longitudinal studies might partly reflect an effect of reverse causality.

### Personality Traits

Personality traits encompass a range of behaviors that can be typically summarized into various patterns or dimensions that are generally stable during adulthood. Different personality traits may be associated with various behavioral and cognitive disorders. A cross-sectional study showed that high agreeableness and openness were correlated with poorer performance in executive function independent of demographics, depression, and cortisol levels (Ouanes et al., 2017). A population-based cohort study of people aged ≥75 years in Stockholm suggested that low neuroticism in combination with high extraversion was associated with the reduced risk of dementia (Wang et al., 2009). A systematic review found that the association of various personality traits with Alzheimer’s disease and dementia was highly consistent across several well-established cohort studies (Terracciano et al., 2014). The population-based surveys and the meta-analysis indicated that certain types of personality traits (e.g., high neuroticism, low conscientiousness, low extraversion, low agreeableness, and low openness) were associated with an increased risk of Alzheimer’s disease and dementia; it was estimated that neuroticism and conscientiousness could account for 13%–11%, respectively, of all Alzheimer cases in the population (Terracciano et al., 2014, 2017b). This suggests that the estimated population attributable fractions for dementia due to these personality traits are generally comparable to those of well-established clinical and lifestyle risk factors such as midlife hypertension, diabetes, and depression (Norton et al., 2014). Finally, personality traits may change along with the development of MCI or dementia such as decreases in conscientiousness and extraversion, and increases in neuroticism (Islam et al., 2019), although a large-scale study showed no evidence for preclinical change in personality prior to the onset of MCI or clinical dementia (Terracciano et al., 2017a). It was proposed that the personality profile for dementia is characterized by high neuroticism and low openness and extraversion, although the personality traits can also be interpreted as an early indicator of subsequent development of dementia (D’Iorio et al., 2018; Yoneda et al., 2018).

### Depression or Depressive Symptomatology

The relationship of depression and depressive symptomatology with dementia has been investigated in numerous studies from a life-course perspective, as previously reviewed (Byers and Yaffe, 2011). Several population-based cohort studies have reported an elevated risk of dementia and cognitive impairment associated with a history of depression (Dotson et al., 2010; Ritchie et al., 2010; Lenoir et al., 2011). A Swedish study of middle-aged women found that self-reported stress in midlife was associated with an increased risk of dementia developed two decades later (Johansson et al., 2010). The meta-analysis of prospective cohort studies reported a double-increased risk for dementia associated with depression (Ownby et al., 2006; Cherbuin et al., 2015), where an increased interval between diagnoses of depression and onset of dementia was associated with an increased risk of subsequent dementia, supporting the notion that depression may be a risk factor for dementia. Although long-term follow-up studies also support a temporal relationship of depression to subsequent cognitive outcomes, it remains debatable regarding whether late-life depression or depressive symptomatology is a preclinical symptom or a causal risk factor for dementia and cognitive impairment (Saczynski et al., 2010; Li et al., 2011). Taken together, earlier-life depression or high depressive symptomatology has often been linked with an over two-fold increased risk of dementia, whereas most population-based cohort studies of late-life depression and dementia risk have supported an association, yet the temporal nature of their association (i.e., whether depression or high depressive symptomatology represents a prodrome or consequence of or a risk factor for dementia) remains to be clarified.

### Lifespan Cumulative Effects of Multiple Intellectual Factors

Evidence from multidisciplinary research has been accumulating that various intellectual or psychosocial factors over the lifespan such as early-life educational attainment and socioeconomic position, midlife greater work complexity, certain personality traits, and more leisure activities in late-life are associated with a reduced risk of cognitive impairment and dementia later in life. Lifespan various intellectual or psychosocial factors are often correlated and their effects may be cumulative. Indeed, several population-based studies using the life-course approaches suggested that cumulative exposures to or composite measures of various cognitive reserve-enhancing factors over the lifespan (e.g., early-life education, midlife work complexity, and late-life leisure activities) were associated with higher cognitive function and a reduced risk of dementia in late-life (González et al., 2013; Dekhtyar et al., 2015, 2016; Wang et al., 2017). The UK Medical Research Council Cognitive Function and Ageing Study suggested that an active cognitive lifestyle (e.g., high education, occupational complexity, and frequent social engagement) in either middle age or late-life was associated with a more favorable cognitive trajectory and a decreased risk of dementia (Valenzuela et al., 2011; Marioni et al., 2012). In addition, the Kungsholmen Project showed that leisure activities containing more than one of the three components (i.e., physical, social, and mental components) seemed to be more beneficial in reducing dementia risk than to be engaged in only one type of activity, suggesting a cumulative effect of multiple activities (Karp et al., 2006). Finally, cognitive reserve capacity could partly mediate the association of leisure-time social, mental, and physical activities with late-life cognitive function (Clare et al., 2017). Notably, we cannot rule out the potential that the observed associations from most of the follow-up studies might be partially affected by unmeasured potential confounders or by reverse causality owing to the long-term latent period (around 20–30 years) of dementia (Jansen et al., 2015; Sajeev et al., 2016).

## Interactions of Genetic Susceptibility With Intellectual Factors

Evidence from epidemiological studies suggested that certain intellectual or psychosocial factors may interact with genetic susceptibility (e.g., APOE ε4 allele) to affect late-life cognitive phenotypes, i.e., the effects of these factors on cognitive phenotypes in aging may vary by genetic susceptibility. For example, several studies have shown that APOE ε4 allele may magnify the risk of dementia and cognitive impairment associated with midlife psychosocial factors such as physical inactivity and cognitive inactivity, such that people with these factors are more vulnerable to dementia and cognitive decline when carrying the ε4 allele (Andel et al., 2008; Carlson et al., 2008; Kivipelto et al., 2008). On the other hand, intellectual or psychosocial factors such as high educational attainment in early life and leisure-time active engagement in physical, social, and mental-stimulating activities later in life could diminish the increased risk of dementia owing to the APOE ε4 allele, even among very old people (Ferrari et al., 2013). In addition, the Singapore Longitudinal Aging Study showed that the association of more leisure activities with a reduced risk of cognitive decline was stronger among carriers of the APOE ε4 allele than non-carriers (Niti et al., 2008). Also, population-based prospective studies showed that cognitive activity and regular physical activity at middle age were associated with a lower likelihood of dementia and poor cognitive performance in later life, in which such protective effect was stronger among carriers than non-carriers of the APOE ε4 allele (Carlson et al., 2008; Chang et al., 2010). Furthermore, several population-based studies have suggested that APOE ε4 allele may modify the association of depression with risk of dementia and cognitive decline, such that people with both depression and the ε4 allele have a markedly increased risk of cognitive decline and dementia (Irie et al., 2008; Niti et al., 2009; Köhler et al., 2010; Kim et al., 2011; Pink et al., 2015). The Finnish CAIDE study of middle-aged cohort revealed that being widowed or divorced from midlife onwards could interact with the APOE ε4 allele to dramatically increase the risk of late-life cognitive impairment and dementia (Håkansson et al., 2009). These studies imply that certain psychosocial factors could interact with genetic susceptibility to affect late-life cognitive phenotypes.

In addition, certain vulnerability personality traits may act interactively with genetic or environmental factors to magnify the deleterious effects of cognitive function in older adults. Data from the Ginkgo Evaluation of Memory study showed evidence that extraversion and neuroticism could potentially modify the association of the APOE ε4 allele with cognitive decline and Alzheimer’s disease, such that the associations of high neuroticism with faster cognitive decline and an increased risk of Alzheimer’s disease were evident only among the ε4 allele carriers and that higher extraversion magnified the deleterious effect of the APOE ε4 allele on cognitive function and the risk of Alzheimer’s disease (Dar-Nimrod et al., 2012). The Victoria Longitudinal Study revealed that the effects of certain personality traits (e.g., neuroticism, extraversion, and openness) on declarative memory were moderated by APOE ε4 allele such that the ε4 allele could magnify the association of lower openness with worse performance in episodic and semantic memory (Sapkota et al., 2016). Similarly, data from this project also demonstrated that the cognitive benefits of complex cognitive activities were greater in non-carriers than carriers of the APOE ε4 allele (Runge et al., 2014). Notably, the Baltimore Longitudinal Study of Aging detected interactions of APOE ε4 allele with openness and agreeableness on the risk of Alzheimer’s disease, in which high openness was protective among the ε4 allele carriers, whereas high agreeableness was protective among non-carriers of the ε4 allele (Terracciano et al., 2014). These studies suggest that the interactions between APOE genotype and personality on cognitive aging may vary by traits and across cohorts. Finally, a follow-up study of middle-aged and older adults (age ≥50 years) suggested that a greater polygenic risk score for Alzheimer’s disease was associated with faster cognitive decline partially *via* higher neuroticism and lower conscientiousness (Stephan et al., 2018). This suggests that the genetic vulnerability for Alzheimer’s dementia may contribute to cognitive phenotypes in part through its association with personality traits. Exploring the interaction of personality traits with genetic susceptibility may help identify older adults with certain characters who are at a substantial risk of accelerated cognitive decline and dementia for early interventions.

## Biological Plausibility: Cognitive Reserve

**Figure 1:**
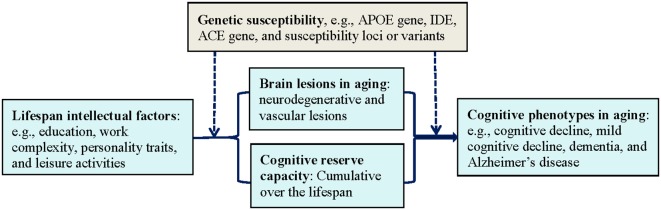
The interplay of lifespan intellectual factors, cognitive reserve capacity, brain pathologies, and genetic susceptibility in cognitive phenotypes of aging. Abbreviations: ACE, angiotensin-converting enzyme gene; APOE, apolipoprotein E gene; IDE, insulin degrading enzyme gene.

Intellectual or psychosocial factors may influence cognitive phenotypes in aging through cognitive reserve and its interplay with overall load of brain pathologies (e.g., microvascular and degenerative pathologies), in which genetic susceptibility may play a part (Figure 1). The concept of cognitive reserve has been proposed to explain the diversities between pathological burden of the brain and clinical phenotypes of cognitive aging (Stern, 2012). More specifically, people with higher cognitive reserve (e.g., higher educational attainments, greater work complexity, and more frequent participation in mental activity) are able to tolerate more pathology in the brain than those with lower reserve prior to expressing cognitive symptoms. This hypothesis is supported by neuroimaging studies, in which the effect of cognitive reserve on the relationship between brain pathology and cognitive function was investigated *in vivo* using neuroimaging markers of neurodegeneration (Brayne et al., 2010; Reed et al., 2010; Murray et al., 2011; Vemuri et al., 2011). Similarly, clinicopathological studies also have shown that cognitive function remains higher in people with a heavier burden of brain degenerative pathology if they also have high education or rich social networks (Bennett et al., 2006; Roe et al., 2007) or have a resilient personality profile (e.g., high conscientiousness and low neuroticism personality traits; Terracciano et al., 2013). Epidemiological evidence also supports the role of cognitive reserve in moderating the longitudinal association between late-life social isolation and poor global cognitive function (Evans et al., 2018). These studies imply that, at a given level of clinical severity of dementia or Alzheimer’s disease, the degree of brain pathology will be greater in individuals with higher cognitive reserve than those with lower cognitive reserve. Of note, there is evidence suggesting that genetic factors (e.g., APOE ε4 allele) and cognitive reserve capacity could interplay to affect cognitive phenotypes in old age, even among very old people, possibly through influencing structural and functional brain networks (Ferrari et al., 2013; Pietzuch et al., 2019).

The underlying neural mechanisms for cognitive reserve are not fully understood. Neural reserve and neural compensation mechanisms have been proposed for cognitive reserve (Stern, 2012). Neural reserve means that cognitive reserve is associated with individual differences in the resilience of pre-existing cognitive networks, whereas neural compensation refers to the idea that some individuals are better than others in using compensatory mechanisms to maintain cognitive function. The concept of cognitive reserve is useful in the clinical assessment of cognitive phenotypes as well as in preventive interventions to delay clinical expression of cognitive outcomes in aging (Qiu and Fratiglioni, 2018).

## Implications for Interventions and Conclusions

Along with rapid global population aging, cognitive impairment and dementia as the major cognitive disorders in aging have posed tremendous economic and societal burden to the modern societies. Epidemiological research has from the life-course perspective provided sufficient evidence suggesting that intellectual factors over the lifespan such as early-life high education, midlife work complexity, and late-life socially-integrative lifestyles (e.g., active engagements in social, physical, and mental activities) may delay the clinical onset of cognitive disorders in aging by providing cognitive reserve, whereas depression or depressive symptoms may confer risk for adverse cognitive outcomes in old age, in which the beneficial or risky effects may vary by genetic susceptibility (e.g., APOE ε4 allele). In this regard, a potential implication of these findings in dementia prevention is to conduct multimodal interventions targeting those intellectual and psychosocial factors together with other modifiable risk factors (e.g., cardiovascular risk factors). However, evidence from randomized controlled trials or intervention studies is still limited. Thus, one of the priorities in future dementia research should be given to testing effects of the multimodal intervention approaches in delaying cognitive decline and the clinical onset of dementia among different populations in the world. The global coordinated effort toward this goal is ongoing, stimulated by the Finnish FINGER multidomain intervention models (see World Wide FINGER Initiative^1^). This approach may help achieve the overall goal of successful aging, especially with regard to healthy brain aging, and thus, may reduce the huge economic and societal burden of cognitive disorders in our aging society.

## Contribution to the Field

As population ages, the age-related cognitive disorders (e.g., MCI or dementia) have posed a serious threat to global public health, health care system, and sustainable economic and societal development. In this narrative review, we summarize the major epidemiological studies from the life-course perspective that investigate the influence of genetic susceptibility (e.g., APOE genotype) and intellectual or psychosocial factors (e.g., education, work complexity, and leisure activities) as well as their interactions on cognitive phenotypes in aging. Population-based studies have suggested that early-life educational attainments, midlife work complexity and social engagements, late-life social, physical, and mental activities, and certain personality traits may affect late-life cognitive phenotypes. Furthermore, certain intellectual or psychosocial factors may interact with genetic susceptibility to affect the phenotypes of cognitive aging such that risk or beneficial effects of these factors on cognitive function may vary by carrying the susceptibility genes. The cognitive reserve hypothesis may partly explain the beneficial effects of lifespan intellectual and psychosocial factors on late-life cognitive function. This implies that, from a life-course perspective, intervention strategies targeting modifiable intellectual or psychosocial factors could interfere with clinical expression of cognitive disorders in old age and delay dementia onset, and thus, may help achieve healthy brain aging and reduce the burden of dementia.

## Author Contributions

CQ and YW contributed to conceptualization and structure of the manuscript, reviewed the literature, and wrote the initial version of the manuscript. YD and JL provided critical comments and revisions. All authors have read and approved the final version of the manuscript.

## Conflict of Interest Statement

The authors declare that the research was conducted in the absence of any commercial or financial relationships that could be construed as a potential conflict of interest.

## Footnotes

^1^www.alz.org/wwfingers/overview.asp#
